# Outcome following valve surgery in Australia: development of an enhanced database module

**DOI:** 10.1186/s12913-017-2002-0

**Published:** 2017-01-17

**Authors:** E. Anne Russell, Christopher M Reid, Warren F Walsh, Alex Brown, Graeme P Maguire

**Affiliations:** 1 Clinical Research Domain, Baker Heart and Diabetes Institute, 75 Commercial Road, Melbourne, VIC 3004 Australia; 2 School of Epidemiology and Preventive Medicine, Monash University, Melbourne, VIC Australia; 3 School of Public Health, Curtin University, Perth, WA Australia; 4 Prince of Wales Hospital, Randwick, NSW Australia; 5 Wardliparingga Aboriginal Research Unit, South Australia Health and Medical Research Institute, Adelaide, SA Australia; 6 School of Population Health, University of South Australia, Adelaide, SA Australia

**Keywords:** Indigenous health, Rheumatic heart disease, Valve surgery, Surgery timing, Outcome indicators

## Abstract

**Background:**

Valvular heart disease, including rheumatic heart disease (RHD), is an important cause of heart disease globally. Management of advanced disease can include surgery and other interventions to repair or replace affected valves. This article summarises the methodology of a study that will incorporate enhanced data collection systems to provide additional insights into treatment choice and outcome for advanced valvular disease including that due to RHD.

**Methods:**

An enhanced data collection system will be developed linking an existing Australian cardiac surgery registry to more detailed baseline co-morbidity, medication, echocardiographic and hospital separation data to identify predictors of morbidity and mortality outcome following valve surgery.

**Discussion:**

This project aims to collect and incorporate more detailed information regarding pre and postoperative factors and subsequent morbidity. We will use this to provide additional insights into treatment choice and outcome.

## Background

Valvular heart disease can be congenital or acquired. Acquired disease can be either a result of aging or due to a disease process that damages valves. Management of valvular heart disease can involve a combination of medication, surgical repair or valve replacement with a mechanical or bioprosthetic valve. There were 9,276 heart valve repair or replacement procedures reported in Australia in the year 2013–14 [1]. From a clinician and patient perspective, the aim is to intervene at a time and in a way that ensures the lowest possible operative complications and mortality with the best short and long-term outcome.

A particular cause of acquired valvular heart disease is rheumatic heart disease (RHD). Whilst now rare in high income countries [2, 3], it remains a condition of global health importance and an important cause of preventable heart disease. In Australia RHD particularly affects Aboriginal and/or Torres Strait Islander peoples (Indigenous Australians) and older non-Indigenous Australians [4, 5].

We have previously analysed data [6] from the Australian and New Zealand Society of Cardiac and Thoracic Surgeons (ANZSCTS) National Cardiac Surgery Database [7] and have identified differences in surgical management for RHD and non­RHD valve disease and for Aboriginal and/or Torres Strait Islander and non­Indigenous Australian patients [5]. In addition we have been able to identify factors associated with outcomes following valve surgery for RHD and non­RHD related valve disease [5, 6, 8].

The details of the Database have been outlined elsewhere [5]. Briefly it is an Australia-wide database for the collection and analysis of the results of cardiac surgery that collates data from Australian hospitals regarding patients who have undergone cardiac surgery, the type of surgery performed, early complications and 30 day mortality. In addition the Database is linked to the Australian National Death Index [9] to assess longer term survival. The Database commenced in 2001 with six surgical centres and currently encompasses 28 Australian sites.

Clinical registries such as this provide a minimum dataset related to the patient, the procedure and outcome. As such they are a valuable resource for informing clinical care, quality assurance activities and for research hypothesis generation. While the Database collects a range of pre-operative patient demographic, co-morbidity and outcome data it does not include prior medication use, detailed echocardiography measurements and non-lethal complications beyond 30 days post-procedure.

The medical management of advanced valvular disease can include anti-platelet and anti-coagulant medication, diuretics, angiotensin-converting enzyme (ACE) inhibitors [10], and beta blockers but it is unclear if these agents can influence early and longer term outcomes following surgery. In addition while valvular disease can be complicated by cardioembolism (e.g., stroke) [10] the influence of such a history prior to surgery on outcome, and how this may influence surgical choice, remains unclear.

Existing studies highlight the importance of echocardiographic assessment of the severity of valve disease and pre­operative valve and heart function. Such data is currently not collected by the existing Database, in particular, left ventricular end­systolic (LVESD) and end­diastolic (LVEDD) diameter and pulmonary arterial systolic pressure (PASP).

Valve morphology has also been shown to predict outcome for those undergoing RHD-related mitral valve repair including the absence of deformity of the mitral valves leaflets and mitral valve prolapse [11] and maintenance of anterior mitral leaflet mobility [12].

Whilst longer-term survival beyond 30 days for the Database is determined from the National Death Index (NDI) [9], other outcomes are currently captured only to 30 days following surgery and only for the surgical site. Non-lethal longer term outcomes relevant to valve surgery include bleeding and thromboembolic complications, heart failure, endocarditis and reoperation.

Thromboembolic events reported in previous studies for follow-up to 7 years following surgery has ranged from none [13] to 5.9% [14] and for up to 10 years, 6% [14] to 24.7% [15] of mechanical valve replacement recipients and 7% [16] to 25% [17] of bioprosthetic valve recipients. Bleeding events have been reported as between 8.8% [17] and 52.6% [18]. The long term risk and burden of heart failure following valve surgery is poorly defined and earlier studies have demonstrated a significant risk of subsequent endocarditis [19] .

Reporting of re-operation varies greatly. After a repaired mitral valve, this has ranged from none at 2 years [20] to 90% at 30 years [21], from 1% [22] at 9 years for all mitral valve replacements, 3.4% [14] at 5 years to 12.6% [23] at 25 years for mechanical valve replacement and 3.6% at 5 years [14] to 63% at 25 years [23] for bioprosthetic valve replacements.

This multicentre, enhanced surveillance system therefore aims to collect short and longer term outcome data to assist in predicting outcomes and providing evidence to inform the development of guidelines to facilitate consistent practice. Utilizing an enhanced data collection system it will collect and incorporate more detailed information regarding pre and postoperative factors at Australian sites that undertake both non-RHD and RHD-related surgery. It will use these more detailed data to provide additional insights into treatment choice and outcome for valve surgery in general and RHD specifically.

Information demonstrated to be important and relevant will be considered for future inclusion in the existing ANZSCTS national cardiac surgery database to assist in predicting outcomes and providing evidence to inform the development of guidelines to facilitate consistent and evidence­based practice in the management of valve disease including for that relating to RHD [10].

## Methods

### Population and method of sampling

Four Australian cardiothoracic surgical sites with significant RHD and non-RHD surgical caseloads representing cases from two different Australian jurisdictions will be included. A random subset of patients having procedures over the preceding ten years, will be chosen from the existing Database.

### Sample size

The sample size will be based on the number required to detect a difference in major adverse prosthesis-related events (MAPE) between bioprosthetic and mechanical valve replacements. MAPE will be defined as a composite outcome of any reoperation, major bleeding, thromboembolic event, or endocarditis during late follow-up [24]. A sample size of 600 patients will be recruited based on an anticipated rate of MAPE over ten years of follow-up of 50% for mechanical valve replacements and 35% for bioprosthetic valve replacement [24], a ratio of mechanical to bioprosthetic mitral valve replacements of 2:1 [5], two-sided alpha of 0.05 and power of 80%.

### Instrumentation

An enhanced baseline dataset with identical definitions for all data points has been created to standardize data collection. Field names and coding have been defined in line with the existing Database data definitions. The enhanced baseline dataset consists of data shown in Table 1. Pre-operative history will be based on linkage with hospital separation data and with reference to the Massive Transfusion Registry (MTR) [25]. Medication and echocardiography data will be ascertained from the index hospital admission for valve surgery including admission history for pre-operative medication, discharge medication and linkage with imaging reporting systems.

**Table 1:** Enhanced peri-operative data collection

Pre-operative history	ICD 10 code [29]
Cerebrovascular diseases	I60-I69
Haemorrhagic	I60-I62
Ischaemic	I63
Transitory ischaemic attack (TIA)	I65-I66
Bleeding	Eye: H21.00, H35.6
	Digestive system: K22.11, K22.8, K25.4, K29.00, K29.01, K29.31, K29.41, K29.51, K29.61, K29.71, K29.81, K29.91, K62.5, K92.0, K92.1, K92.2
	Circulatory system: I85.00, I85.01, I85.11, N93.0, N92.3, N93.9, N95.0, R04.0, R04.1
	Genitourinary system: R04.89, R19.5, R58
	Haemorrhagic disorder due to circulating anticoagulants: D68.3
	I50.9, I50.0, I50.1, I51.5, I11.0, I11.9, I13.0, I13.2
Heart failure	O74.2, O75.4, I97.1, I97.8, I25.5, O29.1, O89.1, I09.81, I27.89
Endocarditis	B33.21, I01.1, I09.1, I33, I38, I39, I42.9
Cardiac surgery (including type)	CABG: 0210 – 0213;
	Valve repair: aortic 02QF, mitral 02QG, pulmonary 02QH, tricuspid 02QJ
	Valve replacement: aortic 02RF, mitral 02RG, pulmonary 02RH, tricuspid 02RJ
	Percutaneous/trans-catheter valve replacement: 623, 628, 637, 634
	Percutaneous valvuloplasty: 38270-01, 38270-02
Arrhythmia	I44.0-9, I45.0-9, I480-9, I49.0-9, I97.8, I47.0, J84.1, M62.8
	Pacemaker and/or defibrillator (insertion but not replacement, removal or adjustment): 38256-00/01 38368-00, 38390-00/01/02, 38350-00, 90202-00/01/02, 38473-00/01, 38470-00/01, 38654-00/03, 38353-00, 38393-00
Pre-operative and discharge	
Medication	Time period
Beta blocker	Pre-operative, on discharge
ACE Inhibitors	Pre-operative, on discharge
Angiotensin Receptor Blocker	Pre-operative, on discharge
Diuretic	Pre-operative, on discharge
Digoxin	Pre-operative, on discharge
Warfarin	Pre-operative, on discharge
NOAC (new oral anticoagulants)	Pre-operative, on discharge
Aspirin	Pre-operative, on discharge
Clopidogrel/Prasugrel/Ticagrelor	Pre-operative, on discharge
Pre-operative echocardiography	Measurement
Left Ventricular End-Systolic Diameter	mm
Left Ventricular End-Diastolic Diameter	mm
Left Atrial Diameter	mm
Left Atrial Area	cm^2^
Pulmonary Artery Pressure (maximal tricuspid regurgitant pressure + estimated right atrial pressure)	mmHg
Valve data	
Mean Gradient	Aortic and mitral valve (mmHg)
Peak Gradient	Aortic valve (mmHg)
Area	Aortic and mitral valve (cm^2^)
Pressure half time	Aortic and mitral valve (ms)
Area Planimetery	Mitral valve (cm^2^)
Jet Area	Mitral valve (cm^2^)
Valve morphology	Aortic and mitral valve
Valve abnormality	Tricuspid and pulmonary valve

In addition to enhanced baseline data collection the project will identify late (more than 30 day) complications potentially associated with valve surgery. In line with pre-surgery morbidity this will be undertaken by using jurisdiction hospital separation data linkage for all hospitalizations and the conditions outlined in Table 1 as well as with reference to the MTR [25]. Outcomes will be recorded and reported according to the *Guidelines for reporting mortality and morbidity after cardiac valve interventions* [24] with comparisons of major morbidity between mechanical and bioprosthetic valves made using MAPE [26].

Once ethics committee approval is obtained for all sites for the data collection, data will be obtained from surgeon and hospital records for the initial admission and entered onto data collection forms. Permission to access the MTR will be sought to identify all valve surgery patients (≥18 years old) who have received at least 5 units of red blood cells (RBCs) within any 4 h time period.

### Analysis plan

The data collected will be analysed using multivariable, logistic and Cox proportional hazard models to identify independent factors associated with the outcome previously analysed [5, 6] and short and long term outcome. This will be undertaken using IBM SPSS Statistics 20 (IBM, New York, USA) and STATA Release 14 (StataCorp LP, Texas, USA). Missing data will be noted and assessed for potential bias. Possible missing data could be specific echocardiographic measurements not performed, which would be missing at random and readmission occurring outside the jurisdiction, which may be not missing at random if they are patients from remote areas. Where the patient’s residential address is determined to be outside the jurisdiction of the surgical site access to local hospital separation data will be sought. Echocardiographic continuous variables will be stratified as necessary for analysis, using acknowledged cut off values (e.g., mild, mod, severe). The analysis will be assessed to determine if the new data is useful for future incorporation in the national ANZSCTS database for ongoing prospective collection.

This study has been reported following the Strengthening the Reporting of Observational Studies in Epidemiology (STROBE) recommendations [27].

## Discussion

The development of this multicentre, enhanced surveillance systems to collect enhanced baseline and longer term morbidity data will aim to assist in predicting outcomes and providing evidence to inform the development of guidelines to facilitate consistent practice. Added to the existing national cardiac surgery database (Australian and New Zealand Society of Cardiac and Thoracic Surgeons (ANZSCTS)) these data may assist in deciding the most appropriate choice and timing of surgery.

Our analysis of the current database [6] has challenged the findings of earlier studies of surgical outcome in other settings. The finding that neither prior nor new post-operative AF was found to be an independent predictor of survival in RHD versus non-RHD valve surgery highlights the importance of considering these conditions as separate entities in the setting of valve surgery [28]. Our earlier finding that poorer preoperative clinical status, based on NYHA class, was also not independently associated with longer term survival requires further investigation with the addition of other cardiac and non-cardiac factors that influence NYHA-measured function to assess an independent effect on survival [6]. The addition of medications, echocardiography results and longer-term follow-up will also assist in strengthening the understanding regarding how pre-operative comorbidities and medication use influence outcome with the ultimate aim of enhance the timing and management of patient with advanced valvular heart disease.

## Conclusion

This article summarises the methodology of a project that aims to collect and incorporate more detailed information regarding pre and postoperative factors and non-lethal outcomes at Australian sites that undertake a significant proportion of RHD and non-RHD surgery. We will use these more detailed data to provide additional insights into treatment choice, timing and outcome.

Information demonstrated to be important and relevant will be considered for future inclusion in an ongoing Australia cardiac surgery registry to assist in predicting outcomes and providing evidence to inform the development of guidelines to facilitate consistent and evidence­based practice in the surgical management of valve disease.

Such data will also be integral to informing future national and international guidelines for the management of advanced valvular heart disease including for RHD as part of the Australian guideline for prevention, diagnosis and management of acute rheumatic fever and rheumatic heart disease [10].

## Abbreviations

ACE: Angiotensin-converting enzyme inhibitors
ANZSCTS: Australian and New Zealand Society of Cardiac and Thoracic Surgeons
LVEDD: Left ventricular end­diastolic diameter
LVESD: Left ventricular end­systolic diameter
MAPE: Major adverse prosthesis-related events
MUHREC: Monash University Human Research Ethics Committee
NDI: National Death Index
PASP: Pulmonary arterial systolic pressure
RBC: Red blood cell
RHD: Rheumatic heart disease
STROBE: Strengthening the Reporting of Observational Studies in Epidemiology
